# The Potential of Ultraviolet Radiation Meters in Secondary Schools as a Sun Protection Intervention Mechanism for Adolescents

**DOI:** 10.3390/ijerph17041137

**Published:** 2020-02-11

**Authors:** Simone Pettigrew, Ashleigh Parnell, Mark Strickland, Rachel Neale, Robyn Lucas

**Affiliations:** 1 Food Policy Division, The George Institute for Global Health, Newtown, NSW 2042, Australia; 2 Kurongkurl Katitjin, Edith Cowan University, Joondalup, WA 6050, Australia; a.parnell@ecu.edu.au; 3 Cancer Prevention & Research, Cancer Council Western Australia, Subiaco, WA 6008, Australia; Mark.Strickland@cancerwa.asn.au; 4 Population Health Department, QIMR Berghofer Medical Research Institute, Brisbane, QLD 4006, Australia; Rachel.Neale@qimrberghofer.edu.au; 5 National Centre for Epidemiology and Population Health, Australian National University, Canberra 2601, Australia; Robyn.Lucas@anu.edu.au

**Keywords:** sun protection, ultraviolet radiation, adolescents, school-based interventions

## Abstract

The aim of this pilot study was to assess whether the installation of ultraviolet radiation (UVR) meters in secondary schools has the potential to improve adolescents’ sun protection-related knowledge, attitudes, and behaviours and reduce their exposure to UVR during school hours. Data were collected from students at two schools via online pre- and post-intervention surveys, measurement of sunscreen usage, polysulfone UVR exposure badges, and photographs of the schoolyards to assess hat and shade use. Several operational issues limited the quantity and quality of data that could be collected, and findings were mixed. While there were no significant changes in either self-reported or observed sun protection behaviours, there were significant improvements in UVR knowledge among students at the intervention school, and reactions to the meter were highly favourable. Students reported consulting the meter regularly and using it to make decisions about their sun protection behaviours. Overall, the study results offer some support for the use of UVR meters in areas frequented by adolescents and provide insights into the process issues that are likely to need to be addressed when attempting to trial sun protection interventions in schools.

## 1. Introduction

Skin cancer is primarily caused by exposure to ultraviolet radiation (UVR) [1], and is highly prevalent in countries such as Australia that have high levels of ambient UVR [2]. Two in three Australians are expected to develop skin cancer by 70 years of age [3], making it critically important to encourage and facilitate higher levels of engagement in sun protection behaviours [4]. The substantial health system burden of skin cancer is such that prevention interventions have been found to be highly cost-effective [5]. As such, further work is needed to inform the development of novel interventions that can expand the suite of available sun protection promotion strategies that can be effective at population and/or specific subgroup levels.

Adolescents are recognised as an especially important target group for sun protection interventions [6,7]. Sun exposure during childhood and adolescence is thought to be of particular importance for skin cancer risk across the life span [1,8]. It is estimated that around 50% of sun exposure up to age 60 years is attained by the age of 20 [9]. Childhood and adolescence are also important times to implement interventions because of the potential to instil positive sun protection behaviours and prevent negative behaviours from becoming entrenched [10].

While the prevalence of use of sun protection behaviours in adolescence varies across different countries [11,12,13,14], overall enactment levels remain suboptimal [14,15,16]. This is especially the case amongst teenagers, with compliance with sun protection recommendations decreasing during adolescence [15,17,18,19]. Teenagers have been described as particularly difficult to target due to reduced parental oversight, a desire to exert independence, increasing concern about appearance, a common desire for a tan, concerns about the opinions of peers, and increased risk-taking, all of which can result in low motivation to comply with sun protection recommendations [13,15,20,21,22]. As a result, the framing and delivery of sun protection messages are particularly important, and direction from authoritative sources may be ineffective if adolescents perceive the communication to be a ‘lecture’ [23]. This has important consequences for intervention design, as behavioural guidance needs to be communicated carefully to minimise psychological reactance.

A further consideration is that while policies have been introduced in many primary schools to ensure children’s exposure to UVR is minimised, this is often not the case in secondary schools [24]. Schools are especially important locations for sun protection interventions because exposure during school hours can account for a substantial part of the total daily exposure to UVR [25]. Students are somewhat different from those in other occupations in that they typically spend their lunch break outside, and hence, are exposed to UVR at peak times [26]. This highlights the need for structural components of school sun protection policies that address the provision of shade and the scheduling of outdoor activities to minimise exposure to harmful levels of UVR [15,25]. In addition, schools are where messages relating to sun protection behaviours are often delivered. Various educational interventions aiming to encourage adolescents to engage in sun protection have been tested in schools: From programs based on increasing knowledge by providing information, through to the use of fear appeals involving simulated sun-induced facial aging (for example, [27,28,29,30,31,32]). These approaches have demonstrated varying levels of success, and typically have a greater impact on knowledge and attitudes than on behaviours. Similarly, analyses of the effectiveness of mass media campaigns targeting adolescents have found high levels of campaign and message awareness [33], but markedly lower levels of compliance with the recommended behaviours [15,16].

Effectively reducing exposure to UVR requires an appreciation of the wide range of factors that influence individuals’ decisions about sun protection [26,34]. In particular, risk awareness, attitudes to recommended risk-avoidance behaviours, perceived barriers, social norms, and perceived self-efficacy in influencing individuals’ behavioural decision making have been noted as important elements of efforts to address the complex range of activities involved in sun protection [26,35,36]. Excessive exposure to UVR often occurs as a result of incidental exposure in contexts where the need for sun protection is not salient because individuals are focused on other activities [37]. Previous research has found that adolescents are receptive to receiving ‘cues to action’ in the physical environment that act as a reminder to engage in sun protection in real time [23].

### Present Study

In Australia, mass media campaigns focusing on increasing awareness of the dangers of skin cancer and the need for sun protection have been aired since the 1980s; some of these have specifically targeted adolescents [38]. As a result, the Australian population has long had a good understanding of the risks of excessive sun exposure [33,39]. It is, therefore, unlikely that lack of knowledge is a major contributor to suboptimal use of sun protection strategies among adolescents in Australia [13,15,16,40]. Alternative approaches are required to supplement existing strategies to provide the impetus for improvements in sun protection behaviours.

It has been noted that it is important to sun protection behaviours to ensure that individuals have access to timely information about UVR in the form of the UV Index (UVI) [26,35,41,42]. The UVI is defined by the World Health Organisation as ‘a simple measure of the UV radiation level at the Earth’s surface and an indicator of the potential for skin damage’ [43]. The UVI was developed to monitor changes in UVR at Earth’s surface resulting from depletion of stratospheric ozone [44], and is now commonly used as an awareness tool for public health purposes. Television, newspapers, websites, and apps are the main methods of communicating the UVI [26,41,42], but these typically require people to access the information actively. A better understanding of how to most effectively disseminate the UVI is needed [35,42].

Given what is known about the inadequate use of sun protection behaviours across the adolescent years, providing information about the UVI in physical locations frequented by adolescents could potentially constitute a cue to action that avoids triggering the psychological reactance that can occur when explicit behavioural instructions are delivered by authority figures. The aim of the present exploratory study was, thus, to assess whether UVR meters can provide a timely, indirect reminder to adolescents that UVR is present at harmful levels, thereby making the need for sun protection salient and potentially stimulating a behavioural response.

## 2. Materials and Methods

Two similar secondary schools located in the same area of Perth, Western Australia were selected for participation in the study, one of which was designated the intervention school and one the control school. The schools were located within 5 kilometres of each other and had similar numbers of students (intervention school—1331 students; control school—1148 students) enrolled across Years 7 to 12 (typically ages 12–18 years). The land area of the intervention school was approximately 8 hectares, and the control school was slightly larger at 11 hectares. Both schools had extensive grassed ovals and open spaces.

### 2.1. The Intervention

The intervention school received a UVR meter in the middle of the study period (Feb–Mar 2019), and the control school was wait-listed to receive a meter at the conclusion of the study.

**Figure 1:**
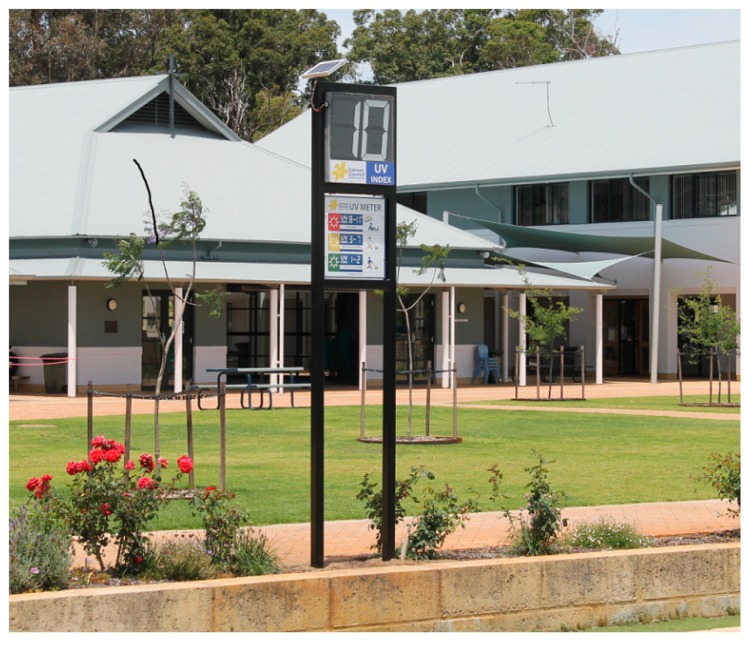
Image of a sun meter with accompanying signage.

**Figure 2:**
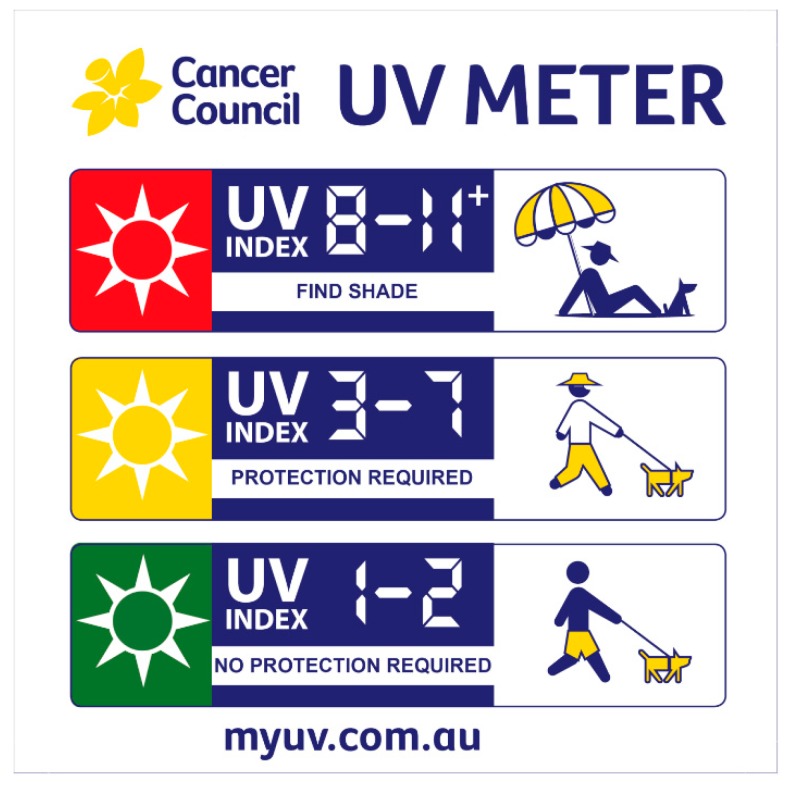
Information sign accompanying ultraviolet radiation (UVR) meter.

A presentation was delivered to students at the intervention school during an assembly on day 10 to coincide with the UVR meter installation. The presentation lasted approximately 15 min and explained the purpose of the UVI and the threshold of ‘3′ as the reading at which the need for sun protection is indicated. Consistent with the recommendation for UVI information to be accompanied by graphical content and actionable messages to provide needed context and increase comprehension [42], a sign featuring a graduated call to action was attached to the meter at the intervention school (see Figure 1 and Figure 2).

### 2.2. Objective Data Collection

Objective data relating to sun protection behaviours (detailed below) were captured over a period of 20 school days across February/March 2019. In an effort to overcome the noted limitations of previous research that has typically relied on self-reported sun-protection behaviours [13,14,15], behavioural data in the form of shade and hat use (via schoolyard photographs), sunscreen use (via the provision of sunscreen bottles), and UVR exposure (via the application of polysulfone badges) were captured objectively. Both schools had two cameras located within school grounds, one in a sunny position and one in a shaded area. Ten photographs were taken at 90-s intervals during a 15-min period in the middle of each lunch break. The cameras were located at a height of approximately three metres. To facilitate analysis, the photographs were viewed on a tiled display comprising 12 full-HD LCD panels, and the Adobe Photoshop (Adobe Inc., San Jose, California, USA) count tool was used to mark each image to enable calculation of the number of students in the shade, in the sun, and wearing hats. Two researchers jointly assessed every image and discussed any differences in coding outcomes to reach consensus.

To objectively assess sunscreen use, both schools were given 20 × 1 litre bottles of sun protection factor (SPF) 30 sunscreen to place in central locations in the schoolyard (1 bottle for each of the 20 days of the study). Schools were provided with digital scales and asked to weigh the bottles of sunscreen at the end of each day and submit the readings at the end of the study period. Each reading was then subtracted from the weight of a full bottle (1082g) to determine the amount of sunscreen used per day at each school.

Polysulfone badges were distributed to the two schools in an attempt to measure students’ exposure to UV radiation. The badges were designed to fit within wristbands worn by the students. In total, 800 polysulfone badges (20 badges × 20 days for both schools) were screened using a spectrophotometer to record the baseline UVR exposure. Both schools were asked to ensure that 20 of their Year 7 students wore the polysulfone badges for each of the 20 days of the study (school hours only). The badges were returned at the end of the study period and screened again using the spectrophotometer (note that the badges have no visible colour change that would alert students to their exposure).

### 2.3. Self-Report Data Collection

Year 7 students were provided with links to the pre- and post-surveys, online, that were live for approximately two weeks prior to and after the 20-day study period. The surveys included items assessing students’ demographic characteristics, participation in a range of sun protection behaviours, and knowledge of the UVI. The post-survey administered to the intervention school additionally included items relating to whether students had seen the UVR meter, their perceptions and use of the meter, and whether they had discussed the meter with others.

The study received approval from the Curtin University Human Research Ethics Committee and the Western Australian Department of Education Ethics Committee (HRE2017-0606). In accordance with ethics clearance requirements, all parents and children were advised that their schools were participating in a study about sun protection. Year 7 children and their parents at both the control and intervention schools were given the opportunity to provide consent for the child to answer the pre and post surveys and wear a polysulfone badge for 20 school days. Only children with both child and parental consent were able to participate. All parents were informed of the locations of the cameras and advised to instruct their children to avoid these areas if they did not want them to be included in any of the photographs.

## 3. Results

The effects of the presence of the UVR meter were assessed in terms of changes in knowledge (survey data), attitudes (survey data), and behaviour (survey and observational data). Relevant outcomes in each domain are outlined below, followed by an account of the forms of data collection that were unsuccessful in generating the intended information.

At the commencement of the study, there were 221 Year 7 students enrolled at the intervention school and 220 at the control school. In total, 157 students completed the pre-intervention survey (n = 77 at the intervention school, n = 80 at the control school, i.e., response rates of 35% and 36%, respectively) and 106 students completed the post-intervention survey (n = 49 and n = 57 at the intervention and control schools, giving response rates of 22% and 26%, respectively). There was a gender skew in the intervention school, with around two-thirds of respondents being female at both time points, compared to approximately half of the students at the control school.

### 3.1. Knowledge

Respondents from the intervention school exhibited significant improvements in awareness of (i) the purpose of the UVI and (ii) the UVI threshold for sun protection. When asked about the name of the weather forecast measure that indicates risk of sunburn (open-ended response item), the proportion of students who correctly identified the UVI increased in both schools, but the change was significant only for the intervention school (17% to 38%, *p* = 0.01 vs. 5% to 14%, *p* = 0.09). Similarly, when subsequently prompted with various weather measure alternatives for the weather forecast measure that indicates risk of sunburn (closed-ended item), there was a significant increase in accurate responses among intervention school respondents (53% to 74%, *p* = 0.02) and a non-significant increase among control school respondents (46% to 62%, *p* = 0.08).

In terms of knowledge of the UVI threshold, the proportion of intervention school respondents correctly nominating a UVI of 3 as the indicator for risk of sunburn almost doubled over the study period (29% to 56%, *p* = 0.02), while the change among control school students was non-significant (9% to 12%, *p* = 0.73). When asked whether they had seen the UVR meter at their school, 93% of the respondents (42 of the 45 students who answered this question) from the intervention school answered ‘Yes’.

### 3.2. Attitudes

Table 1 presents the results of the semantic differential scales relating to the intervention school respondents’ perceptions of the UVR meter. Attitudes to the meter were favourable, with the mean values for all assessment criteria being above the mid-point of 3 on the five-point scale. Large majorities reported that the meter was useful, important, informative, convenient, and worthwhile. More moderate outcomes were obtained on the criteria of being innovative and interesting, and the least favourable aspect of the meter was considered to be its attractiveness.

### 3.3. Self-Reported Behaviours

All of those who reported seeing the meter, also reported looking at the UVI. Most indicated that they looked at the UVI reading on the meter at least once per day, and more than one-third reported looking at it multiple times per day (see Table 2).

Table 3 presents the results relating to the reasons selected by respondents for consulting the UVR meter. Of the four response options relating to recommended sun protection strategies (wear a hat, use sunscreen, stay in the shade, and stay indoors), the most commonly reported purpose for viewing the UVR meter was to decide when to go into the shade (71% selecting ‘Sometimes’ or ‘Often’) and the least commonly reported purpose was to decide when to wear a hat (40%).

In terms of sun protection behaviours enacted during lunch breaks, the most commonly reported strategy among both intervention and control respondents was staying in the shade. As shown in Table 4, there were no significant differences in self-reported sun protection behaviours during lunch breaks between the intervention and control school respondents, either at baseline or at the end of the study period.

### 3.4. Observed Behaviours

Table 5 presents the photograph results relating to the numbers of students in shaded areas, in the sun, and wearing hats in the intervention and control schools across the study period. The lens of the camera in the sunny area at the control school was smudged with what appeared to be a smear of sunscreen on the first day of the intervention, causing the photographs to be too blurry to analyse. This was not detected until the cameras were retrieved at the end of the intervention period, resulting in a substantial loss of data. Only photographs taken in the shaded areas of both schools were assessed to ensure comparability (n = 200 photographs for each school).

There were no significant changes in sun protection behaviours observed in either school. In the case of the intervention school, this lack of significant change was likely to be at least partially attributable to the very high proportion of students within the photographed area who stayed in the shade at baseline (92%). Shade use was lower among students at the control school in both periods, however, much less shade was available within the photographed area for this school. Hat use was very low at both schools during the pre- and post-intervention periods.

### 3.5. Unsuccessful Behavioural Data Collection Methods

Two key outcome measures were unable to be adequately assessed due to data collection difficulties. These related to sunscreen use and measurement of UVR exposure using polysulfone badges.

**Figure 3:**
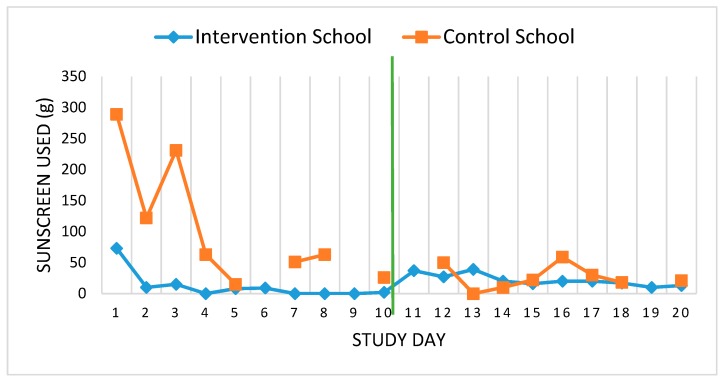
Average sunscreen use per day. Note: Intervention period commenced on day 11.

In terms of sunscreen usage, results were not collected at the control school for four of the 20 days of the study period, preventing meaningful comparisons. As shown in Figure 3, sunscreen use peaked during the first three days of the study period when much higher usage levels were reported, perhaps due to the novelty factor of sunscreen being available in the schoolyard. Both schools exhibited changes in average daily sunscreen use between the pre- and post-intervention periods, but in different directions. The average amount of sunscreen used at the intervention school increased from 11.7 g per day pre-intervention to 21.9 g post-intervention, while usage rates decreased over time in the control school from 107.5 g to 26.2 g per day. However, in both schools the average amount used was negligible in both the pre- and post-intervention periods. The recommended amount of sunscreen used for one full body application is about 35 g [45], meaning that the average amount used per day by the end of the intervention was less than one application.

Missing data problems were more pronounced for the polysulfone badges. Due to a combination of non-compliance and possible user issues (e.g., badges being covered by shirt sleeves), a large proportion of the polysulfone badge data had to be treated as missing data. Overall, 263 (33%) badges were returned with readings of zero, with missing data rates substantially higher in the post-intervention period compared to the pre-intervention period (25% vs. 42%). The intervention school did not record instances of where badges were not worn by students, which prevented assessment of whether nil values related to lack of sun exposure or non-wear. Due to the large amount of missing data, the badge readings were not analysed.

## 4. Discussion

New ways of encouraging adolescents to use sun protection strategies are needed to overcome substantial barriers to use that are resulting in highly suboptimal levels of sun protection enactment [21,22,42]. The present study was novel in its aim of testing the potential efficacy of UVR meters in schools and including objective methods of data collection to assess the extent and nature of any intervention effects. Overall, the results indicate that adolescents may view UVR meters as appropriate reminders of the need for sun protection, with four-fifths of the post-intervention sample reporting that they found the meters to be useful, informative, important, and convenient. However, given the lack of improvement in both self-reported and observed sun protection behaviours, it appears that the meter may have been merely reinforcing positive behaviours among those already using them. This suggests that additional strategies would be needed to convert increased awareness of the need for sun protection at a given point in time to actual behavioural change among those who are failing to comply with recommendations.

The study outcomes are consistent with systematic reviews that have reported mixed or disappointing results relating to the effects of disseminating UVR radiation information to the public [35,42,46]. Knowledge is an important, but typically insufficient, precursor to behaviour change [46]. Sun protection interventions targeting adolescents thus need to be multi-component to address the various individual, social, and environmental factors that combine to influence sun protection behaviours [32,34,47]. For example, it has been suggested that the outcomes of school-based interventions may be amplified if: (i) They are supported by formalised sun protection policies in schools [48]; (ii) parents are included as target audiences [47]; and (iii) the physical environment is made as conducive as possible, such as through the provision of effective shade structures [25,49]. In addition, mass media campaigns are likely to continue to play an important role in ensuring favourable social norms relating to the enactment of sun protection behaviours [15,50]. In the context of this broad suite of initiatives, the location of UVR meters in places frequented by adolescents appears to be a potentially worthwhile means of supplementing other skin cancer prevention strategies in a manner that is low cost and highly acceptable to the target audience.

Further efforts could also be made to assess the efficacy of UVR meters combined with other skin cancer prevention strategies at the total population level. Research conducted in holiday contexts where adolescents spend large periods of time outdoors has indicated the importance of UVR awareness [23,51], suggesting that the provision of real-time UVR information in the form of UVR meters could be beneficial in a wider range of locations.

Recent research suggests that a UVI threshold of 3 for starting sun protection may not be appropriate, with a more nuanced message incorporating both intensity (UVI) and duration of exposure required [52,53]. This more complicated message could still be supported by the UVR meter, but would require additional messaging around the duration. The risks and benefits of changing and complicating the current simple message of using sun protection when the UVI is 3 or greater would need to be carefully considered.

### Study Limitations and Strengths

This was an exploratory study involving only one intervention school and one control school, and as such further research is needed to determine whether the results are likely to apply to students attending schools in other parts of Australia and beyond. The constraints of an opt-in participation process that required written permission from both parents and children resulted in a modest sample among the Year 7 survey respondents. The ability to include all adolescents appearing within the camera field enabled a more comprehensive assessment of sun protection behaviours across year groups, although the apparent sabotaging of one of the cameras effectively halved the amount of observational data that could be captured through this means. Future intervention studies may consider the placement of cameras in less accessible locations to prevent similar problems.

In addition to the sample size, major limitations of the present study included the short intervention period (only 10 school days) and the failure to collect reliable UVR exposure information via the use of polysulfone badges. A longer study period may be required to enable intervention effects to manifest, although it is also possible that the most pronounced effects would be immediate, due to the novelty of the meter, and that decay in any achieved behaviour change could occur over time. The use of dosimeters with adolescents is recognised as challenging [15,50,53], and the experiences in this study support the recognised need to establish effective communication with individuals at multiple levels within participating schools to optimise protocol implementation [32].

A strength of the present study was the focus on the effects of UVR meters as a source of UVR information without the confounds associated with this information being delivered in conjunction with other weather-related information. It has been noted that a limitation of previous research has been the inability to separate out the effects of the UVI component of interventions testing the effectiveness of other dissemination methods, such as television broadcasts, websites, and apps [35]. Further, the present study combined self-report and observational methods of data collection to overcome the reliance in previous research on self-reported enactment of sun protection behaviours among adolescents [13,14,15].

## 5. Conclusions

Suboptimal levels of sun protection among adolescents highlight the need to identify alternative intervention options to supplement existing skin cancer prevention strategies. The results of this exploratory study indicate that the provision of salient, real-time UVI information in schools may constitute a viable and acceptable means of ensuring adolescents have the information they require to make informed decisions about sun protection. While there were no significant changes in either self-reported or observed sun protection behaviours, there were significant improvements in UVR knowledge among students at the intervention school, and reactions to the meter were highly favourable. Students reported consulting the meter regularly and using it to make decisions about their sun protection behaviours. The study results support the use of UVR meters in areas frequented by adolescents if implemented in combination with other strategies as part of a comprehensive approach to improving adolescents’ sun protection behaviours.

## Figures and Tables

**Table 1:** Perceptions of the UVR meter (n = 42 students at the intervention school who reported seeing the UVR meter).

Scale Anchors	Mean	*n* ^1^	% ^1^
Useless/Useful	4.38	33	79
Unimportant/Important	4.33	33	79
Uninformative/Informative	4.29	34	81
Inconvenient/Convenient	4.26	34	81
Not worthwhile/Worthwhile	4.12	30	71
Traditional/Innovative	3.93	27	64
Boring/Interesting	3.74	25	60
Unattractive/Attractive	3.29	18	43

^1^ Selected 4 or 5 on a five-point scale from 1 (Useless, Unimportant, etc.) to 5 (Useful, important, etc.).

**Table 2:** Viewing and discussing the UVR meter (n = 42 students at the intervention school who reported seeing the UVR meter).

Outcome variables	*N*	%
**Frequency of viewing the meter on an average day:**		
Multiple times a day	16	38
About once a day	19	45
Less than once a day	7	17
Never	0	0
**Others with whom the meter was discussed:**		
Friends	25	60
Teachers	21	50
Family members	18	43
Other	15	36

**Table 3:** Reasons for viewing the UVR meter (n = 42 students at the intervention school who reported seeing the UVR meter).

Selected reasons	Mean	*n* ^1^	% ^1^
Check the UVR index	2.93	31	74
Decide when to go in the shade	3.00	30	71
Learn about how/when the UVR index changes during the day	2.64	25	60
Decide when to wear sunscreen	2.64	24	57
Decide when to go inside	2.50	22	52
Decide when to wear a hat	2.29	17	40

^1^ Selected 3 or 4 on a four-point scale of 1 (Never) to 4 (Often).

**Table 4:** Self-reported sun protection behaviours during lunch breaks.

	Intervention School							Control School						
	Pre *n* = 77			Post *n* = 49			Δ in Mean	Pre *n* = 80			Post *n* = 55 *			Δ in Mean
Strategy	Mean	*n* ^1^	% ^1^	Mean	*n* ^1^	% ^1^		Mean	*n* ^1^	%^1^	Mean	*n* ^1^	% ^1^	
Stay in the shade	3.71	51	66	3.94	35	71	0.23	3.93	58	72	3.80	39	71	−0.13
Use sunscreen	2.97	27	35	3.00	17	35	0.03	3.16	33	41	3.29	25	45	0.13
Wear a hat	2.64	23	30	2.33	10	20	−0.31	3.06	31	39	2.69	12	22	−0.37
Stay indoors	2.84	21	27	2.98	15	31	0.14	3.01	28	35	3.24	28	51	0.23
Wear clothes that cover your legs	2.58	16	21	2.37	10	20	−0.21	2.33	12	15	2.22	3	5	−0.11
Wear sunglasses	1.91	9	12	1.67	5	10	−0.24	2.23	14	18	2.07	6	11	−0.16
Wear clothes with long sleeves	2.34	13	17	2.16	9	18	−0.18	2.02	6	8	2.04	1	2	0.02

^1^ Selected 4 or 5 on a five-point scale of 1 (Never) to 5 (Always). * 2 students did not complete the questions on sun protection behaviours.

**Table 5:** Sun protection behaviours during lunch breaks.

	Intervention School					Control School				
	Pre		Post		Δ in %	Pre		Post		Δ in %
Average Number of Students Per Lunch Break:	*n*	%	*n*	%		*n*	%	*n*	%	
In photographed area	37	-	38	-	-	13	-	12	-	-
In shade	34	92	36	93	3	9	69	8	67	-2
In sun	3	8	3	7	0	4	31	4	33	2
Wearing a hat	1	3	1	3	0	<1	<1	<1	<1	0
